# Docetaxel-loaded exosomes for targeting non-small cell lung cancer: preparation and evaluation *in vitro* and *in vivo*

**DOI:** 10.1080/10717544.2021.1951894

**Published:** 2021-07-15

**Authors:** Ying Wang, Mimi Guo, Dingmei Lin, Dajun Liang, Ling Zhao, Ruizhi Zhao, Yan Wang

**Affiliations:** a School of Chemistry and Chemical Engineering, Guangdong Pharmaceutical University, Guangzhou, China; b School of Traditional Chinese Medicine, Guangdong Pharmaceutical University, Guangzhou, China; c The Second Affiliated Hospital of Guangzhou University of Chinese Medicine, Guangzhou, China

**Keywords:** Exosomes, anticancer, docetaxel, targeted drug delivery, NSCLC, *in vitro*, *in vivo* imaging

## Abstract

Non-small cell lung cancer (NSCLC) is a highly lethal disease and the majority of NSCLC patients are desperate for therapies that can effectively target their cancer and ultimately improve their overall survival. Docetaxel (DTX) represents the first-line of the antitumor agent that is used to treat NSCLC; however, it has poor solubility in water and unsatisfactory encapsulation efficiency. In our study, exosomes were isolated from A549 cancer cells by ultracentrifugation and then characterized using transmission electron microscopy (TEM), nanoparticle tracking analysis (NTA), and Western blot (WB). The particle size changes of EXO and EXO-DTX were measured daily for seven days to test the stability. DTX was selected payload by electroporation (EXO-DTX). For the *in vitro* evaluation, cell proliferation, cell cycle, cell apoptosis, reactive oxygen species (ROS) assay and cellular uptake were evaluated in the A549 cells. Also, this study evaluated the target and therapeutic effect of DTX as an antitumor agent *in vivo*. As a result, EXO-DTX with a particle size of 149.5 nm were successfully prepared and the cytotoxicity of the EXO-DTX was much greater than that of DTX monomers. Exosomes significantly increased the cellular uptake *in vitro* evaluation and showed better targeting to tumor tissue compared to the free DTX in the mice. We also explored the potential of tumor cell-derived exosomes as a drug delivery agent to target the parent cancer. Hence, we conclude that exosomes might be used as a potential antitumor drug delivery system (DDS).

## 1. Introduction

Lung cancer is the main reason for cancer-associated mortality in the world (Bar et al., 2019). Since it is diagnosed and treated in the advanced stages, it rapidly metastasizes and shows a high recurrence rate. Despite a noteworthy number of therapeutic methods being constructed to combat lung cancer, non-small cell lung cancer (NSCLC) remains a harmful tumor, especially with a low survival rate (Altai et al., 2018). Numerous studies have been carried out to improve patient survival and reduce patient suffering. Currently, most patients prefer surgery for NSCLC (Chang, 2011). However, a small number of patients are ultimately suitable for surgical excision.

Through hyaluronic acid-mediated CD44 targeting (Xu et al., 2020), liposomes that specifically deliver ICG to NSCLC cells are manufactured to achieve a therapeutic effect. However, hyalurosomes are only effective for photosensitizers, and CD44 modification is needed to achieve the target, which greatly limits its application. Light response targeted therapy (Jaque et al., 2014) for NSCLC has been attracted much attention due to its excellent efficacy and low toxicity. However, its treatment requires laser irradiation of specific wavelengths, which is very inconvenient and the patient's compliance is poor. Although currently available targeted therapies, such as gefitinib and alfa, are targeted specifically for the epidermal growth factor receptor (EGFR) pathway in NSCLC, drug resistance is inevitable (Wei et al., 2019). Therefore, new therapies are urgently needed to address the problem of targeting and drug resistance.

Docetaxel (DTX) (Yang et al., 2017) has been found to have a great potential therapeutic effect in NSCLC treatment. However, unsatisfactory water-solubility and side effects have lowered the distribution of selective DTX *in vivo*, limiting their further clinical applications (Chen et al., 2019). Thus, it is necessary to develop a new DTX agent to solve the problem of the side effects of distribution in the body in NSCLC treatment.

Exosomes as targeted drug delivery systems (DDSs) have become attractive in the field of drug delivery of a chemotherapeutic drug. Exosomes were reported to hold many RNA components and carry them to the target cells, acting as a driver (Valadi et al., 2007). For the permeability effect, nanoparticles ranging from 20 to 200 nm may be used, which can easily leak into the tumor region and retain themselves for a long time (Wu et al., 1993; Yu et al., 2013). Formed during the late budding of the nucleosome, exosomes develop into intracellular polyvesicular nucleosomes that contain nucleic acids and proteins (Al-Nedawi et al., 2008; Skog et al., 2008; Trajkovic et al., 2008; Demory Beckler et al., 2013; Kahlert et al., 2014), including the members of the membrane-spanning protein family (CD9, CD63, and CD81) (Taylor & Gercel-Taylor, 2011). The surface of most cancer cells was observed to usually overexpress certain receptors (Temming et al., 2005; Byrne et al., 2008; Danhier et al., 2010). Exosomes may be adopted as a carrier to deliver the antitumor drugs (Kibria et al., 2018; Qiao et al., 2020). Paclitaxel (PTX), a potent chemotherapeutic agent, was also previously loaded on U-87 cell-derived exosomes (Salarpour et al., 2019). Batrakova (Batrakova & Kim, 2015) has proven that exosomes could be used in the drug efficient targeted therapy and drug delivery approach. Yang et al. (2020) developed a novel integrated microfluidic device, which can isolate and detect lung cancer-specific exosomes *in situ* from the urine of lung cancer patients with high sensitivity and specificity. Exosomes come from cells themselves, and as carrier materials, they will not produce toxic and side effects on the body (Li et al., 2020). They do not need to be modified by specific proteins, nor do they need specific drug administration methods, so they can achieve the role of targeted therapy and have a good development prospect. Moreover, it has good biocompatibility. The autologous exosomes can reach the mother cells more specifically and play a targeted role.

**Figure 1.**
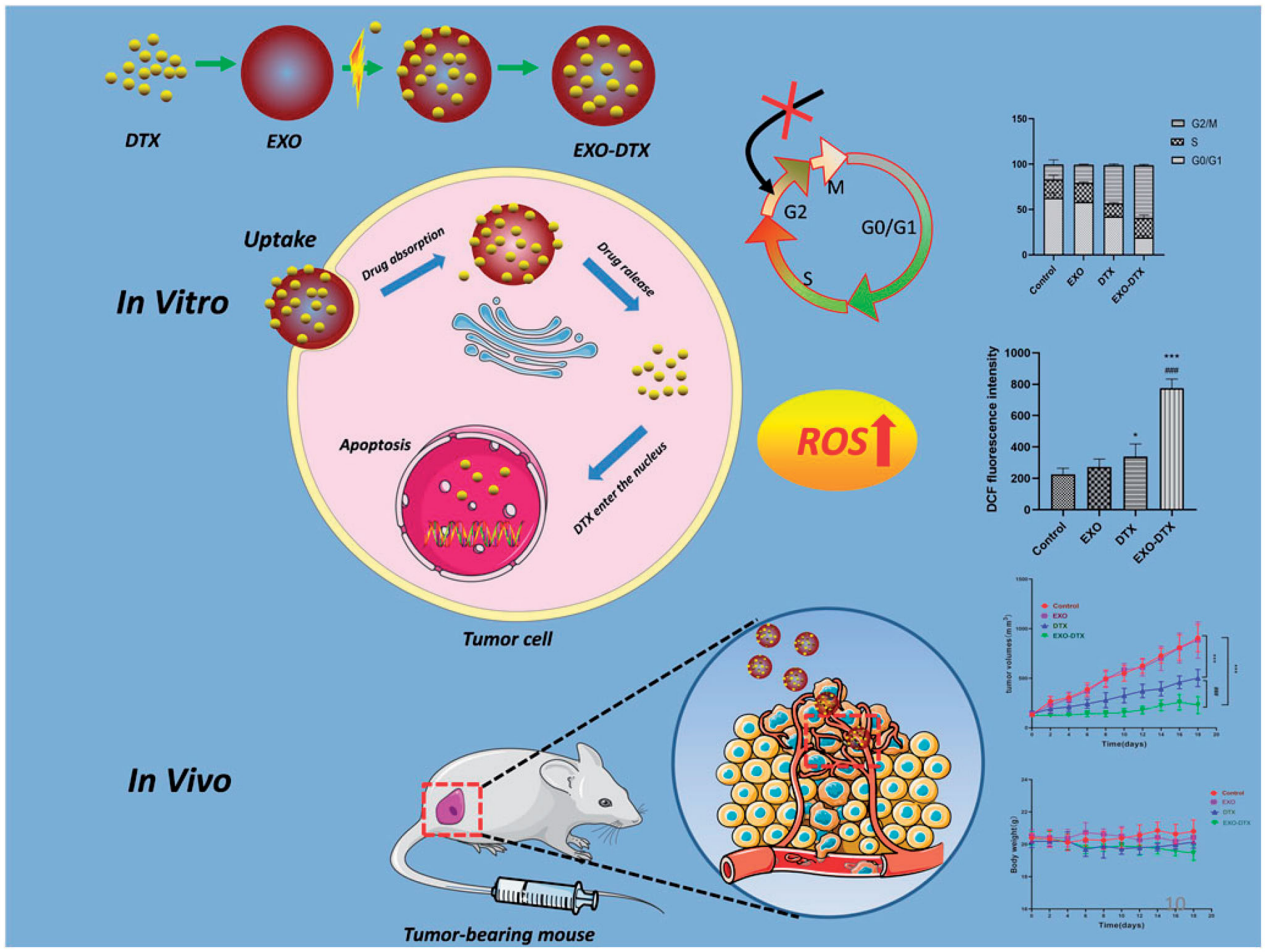
Schematic diagram of the action of EXO-DTX.

In this study, A549-exosomes were prepared, and their effects were evaluated in the A549 cells *in vitro* and *in vivo*. Cellular uptake of the exosomes was assessed using Image J (Bethesda, MD). Finally, the A549 tumor models were constructed, and the tumor-targeting exosomes were examined *in vivo* using a near-infrared fluorescence imaging system. Overall, our results indicated that the proposed EXO-DTX DDS could be adopted as an efficient short-run treatment for NSCLC (Figure 1).

## 2. Materials and methods

### 2.1. Materials

Docetaxel (≥99%) and Cy5.5 were purchased from Nantong Feiyu Biotechnology Co., Ltd. (Nantong, China), while the Cell Counting Kit-8 (CCK-8) was procured from Dojindo (Kumamoto, Japan). The apoptosis assay kit was purchased from KeyGen Biotech (Nanjing, China). The cell cycle assay kit was purchased from Invitrogen (Carlsbad, CA), coumarin-6 (C6) was purchased from Aladdin Reagent Co. (Shanghai, China). Acetonitrile and distilled water (HPLC grade) were obtained from Wenrui Ltd. (Guangzhou, China).

A549 cells were obtained from the ATCC (Manassas, VA). All the reagents were provided by Yike, Life Technologies (Carlsbad, CA). The cells were incubated at 37 °C in a 5% CO_2_ incubator.

BALB/c nude mice aged between 3 and 4 weeks were purchased from Guangdong Animal Center (Guangzhou, China). As per the directions released by the China’s Council on Animal Care (room temperature, 20–22 °C; relative humidity (RH), 50–60%), the animals were raised with free access to water and standard laboratory feed. The experiments were carried out in compliance with the guidelines issued by Guangzhou Pharmaceutical University.

### 2.2. Exosome isolation

The exosomes were isolated from the A549 cells by ultracentrifugation (Théry et al., 2006). First, the cells were cultured in the T75 flasks and incubated until confluent. Later, the medium containing exosomes was harvested (conditioned medium) at the preset timing. The dead cells, debris, and microvesicles were separated from the cells using the differential centrifugation method (300×*g*, 10 min; 2000×*g*, 10 min; 10,000×*g*, 30 min). The exosomes were then pelleted out and washed once with PBS using ultracentrifugation (100,000×*g*, 70 min). Finally, the pellet was resuspended in PBS, followed by filtration using a 0.22 µm syringe filter. The sample was preserved at −80 °C until subsequent analysis.

### 2.3. Characterization of exosomes

Exosomes were characterized using the following methods: (1) transmission electron microscopy (TEM) was used for characterization of exosomes, while (2) nanoparticle tracking analysis (NTA) and (3) Western blotting (WB) assay were used to verify the A549-EXO antigens (ALIX, CD9, and CD63). A Zetasizer Nano ZS (Malvern Instruments, Malvern, UK) was used to determine the polydisperse index (PDI) and zeta potential of the exosomes. Also, the BCA protein assay kit was utilized to measure the overall content of exosome-derived proteins (Savina et al., 2002).

### 2.4. Preparation of EXO-DTX

The EXO-DTX was prepared based on the pores formed on the membranes following an electrical signal stimulation. Alvarez-Erviti et al. (2011) were the first to successfully load siRNA into EVs through electroporation, where 2 mg (protein concentration) of exosome was mixed with 120 µg of DTX dissolved in DMSO. This mixture was then used for electroporation: five mild electric shocks at 0.75 V in 15 s intervals were applied using a Gene Pulser II Electroporator (Bio-Rad, Hercules, CA) to load the DTX into the EXO.

### 2.5. Characterization of EXO-DTX

In order to prove that the loading of the DTX was successful into the EXO, we characterized EXO-DTX using TEM, NTA, WB (Cheng et al., 2019), PDI, and zeta potential. Besides, to investigate the stability, EXO and EXO-DTX were placed at 4 °C and their particle size changes were measured daily for seven days.

### 2.6. The encapsulation efficiency and drug-loading of DTX on exosomes measured using HPLC

The encapsulation efficiency (EE, %) and drug-loading of DTX loaded into the exosomes were measured using HPLC (Saari et al., 2015). First, 0.5 mL of EXO-DTX was added to the Super filter and then centrifuged at 10,000×*g* for 10 min to remove the excess free drug. The supernatant containing the free drug was collected and filtered through a 0.22 µm filter, which was then measured using HPLC. The EE (%) and drug-loading of the drug were determined by dividing the real drug content within micelles by the overall drug amount within the solution. The following formula was utilized to calculate the EE (%) of the DTX:

$$\text{Encapsulation efficiency }(\%)=\frac{M_{1}-M_{2}}{M_{1}}\times 100$$

where $M_{1}$ and $M_{2}$ are the initial amount of added DTX and free DTX, respectively.

### 2.7. The study of *in vitro* drug release

Dynamic dialysis (Zambito et al., 2012) was utilized to examine the release behaviors of EXO-DTX and DTX *in vitro*. First, 4 mL of EXO-DTX and DTX dispersion was added to the dialysis membrane tube, which was soaked beforehand (*M_W_* threshold: 8000–14,000 g/mol). To this, 200 mL of PBS was added. This was followed by the placement of dialysis bags in the centrifugal tube (containing normal tissues environment and simulated tumors environment were at pH 7.4 at 37 ± 1 °C and pH 5.8 at 42 ± 1 °C) Then, 2 mL of fresh pre-heated medium was used to replace 2 mL of incubation medium at 0.5, 1, 2, 4, 8, 12, 24, 36, and 48 h, respectively. The dissolution medium was taken at predetermined time points and analyzed by HPLC.

### 2.8. *In vitro* anti-cancer activity assay

#### 2.8.1. Cytotoxicity assay

The cytotoxicity was determined by the CCK-8 assay. A549 cells (1 × 10^4^/well) were seeded into the 96-well plates and incubated for 24 h at 37 °C, followed by 24/48/72 h of DTX, EXO, or EXO-DTX treatment at a specific dose. After removing the medium, 100 µL of CCK-8 solution was added to all the wells (10%) and incubated for another 30 min. At last, the absorbance (OD) value was detected at 450 nm using the microplate reader.

$$\text{Cell viability \% }=[(O_{m}-O_{b})/(O_{u}-O_{b})]\times 100$$

where $O_{m},$ $O_{u},$ and $O_{b}$ stand for OD values in drug treatment, non-drug treatment, and blank groups, respectively.

#### 2.8.2. Cell apoptosis assay

A549 cells (5 × 10^5^/well) were seeded into the six-well plates and incubated for 24 h at 37 °C. After incubating six-well plates for 24 h, the A549 cells were further treated with EXO, DTX, or EXO-DTX for an additional 24 h. Later, the mixture was centrifuged for 10 min at 1000 rpm, and the cell suspension was collected. After the corresponding treatments, the apoptotic cell proportions were analyzed using the Annexin V-FITC staining. Finally, the samples were analyzed using flow cytometry (FACS).

#### 2.8.3. Cell cycle assay

A549 cells (5 × 10^5^/well) were seeded into the six-well plates and incubated for 24 h at 37 °C, and then treated with EXO, DTX, or EXO-DTX. After 24 h of EXO, DTX, and EXO-DTX treatments, we harvested A549 cells for FACS analysis. Cell proliferation was indicated by the cell proliferation index (PI), calculated as follows: PI=(S + G2/M)/(G0/G1 + S + G2/M)×100%, where different letters stand for different cell proportions at various cell cycle stages.

#### 2.8.4. Transwell assay

This assay was carried out in an 8-µm transwell chamber. First, 100 µL of Matrigel (1:40, 50 mg/L) was added into the upper transwell chamber. Thereafter, 100 µL of cell suspension (containing 2 × 10^5^ cells) was further added to the upper chamber, while the lower chamber was pipetted with 600 µL of DMEM. After 24 h of incubation, methanol was added to fix the cells for 10 min while 1% crystal violet staining solution was added to stain the cells. Later, eight fields of view (FOVs) were chosen at random to count the cell numbers under the microscope. The experimental steps of the cell migration assay were identical to those of the invasion assay, except that there was no Matrigel added beforehand. For each group, three independent wells were used.

#### 2.8.5. Intracellular reactive oxygen species (ROS) measurement

After the 96-well plates were incubated for 24 h, EXO, DTX, or EXO-DTX was added to A549 cells for another 24 h. Then, the cells were stained using DCFH-DA for 20 min at 37 °C, which was recorded by a laser confocal scanning microscope at ×20 magnification.

### 2.9. Cellular uptake *in vitro*

#### 2.9.1. Cellular uptake in autologous cells

The *in vitro* cellular uptake of EXO-C6 (Zhang et al., 2020) was investigated in the A549 cells. A549 cells (1 × 10^4^/well) were seeded into the 96-well plates for a period of 24 h. Later, the cells were incubated using free C6 or EXO-C6 for 1/2/4 h at 37 °C. After incubation, the cells were rinsed with PBS, followed by fixation for 15 min using 4% paraformaldehyde (PDA) and then staining for 5 min using 4**′**,6-diamidino-2-phenylindole (DAPI). Finally, the fluorescence signals were observed using a fluorescent microscope.

#### 2.9.2. Cellular uptake in heterologous cells

To compare the autologous and heterologous cellular uptake of exosomes, A549 cells, H1975 cells, and HepG2 cells were cultured. Later, the cells were incubated using EXO-C6 for 1/2/4 h at 37 °C. After incubation, the cells were rinsed with PBS, and then fixed for 15 min using 4% PDA and stained for 5 min using DAPI. Finally, the fluorescence signals were observed using a fluorescent microscope, and fluorescence intensity was analyzed using Image J.

### 2.10. *In vivo* anti-cancer activity assay

#### 2.10.1. Evaluation of anti-cancer effect and imaging analysis *in vivo*

The A549 xenograft mouse model was built by subcutaneously injecting 100 µL of A549 cell suspension (1 × 10^7^ cells/mL) in the left legs of the mouse. To gain an insight into the EXO-DTX uptake sites *in vivo*, we used Balb/c nude mice (3–4 weeks old, 20–25 g). EXO-DTX labeled with the fluorescence probe Cy5.5 (named as Cy5.5-EXO-DTX) (Doleschel et al., 2012; Yoo et al., 2012) were injected into the A549 tumor-bearing mice. Later, the IVIS imaging system was employed to monitor the Cy5.5-EXO-DTX biodistribution. After the A549 tumor grew to about 120 mm^3^ in size, the tumor-bearing mice were randomized into the following four groups: (1) normal saline (NS), (2) Cy5.5-EXO, (3) Cy5.5-DTX (4 mg/kg equivalent), and (4) Cy5.5-EXO-DTX (4 mg/kg equivalent). The mice were then imaged at 1, 2, 4, 8, 12, and 24 h (*n* = 6) after the administration of probes. The hearts, livers, kidneys, lungs, spleens, and tumors of the mice were collected after 4 h. The fluorescence distribution of the isolated organs was then observed with an *in vivo* imaging system. The bodyweight of mice and their tumor volume were recorded after an interval of two days. Specifically, the following equation was used to measure the volume of the tumor:

$$\text{Tumor}\text{volume}=0.5\times {\left(\text{width}\right)}^{2}\times \left(\text{length}\right).$$

#### 2.10.2. Evaluation of the *in vivo* toxicity of EXO-DTX

To evaluate the treatment-induced toxicity in the vital organs, all animals were killed after 20 days of treatment, and at once, their tumor tissues and vital organs (liver, heart, spleen, lung, and kidney) were dissected. Later, the vital organs were fixed with 4% PDA, sectioned, and then stained with H&E.

### 2.11. Statistical analysis

All data points on the graph from three independent experiments were represented as mean ± SD. Statistical analyses (one-way ANOVA) were performed using GraphPad Prism 8 (La Jolla, CA). The differences between the two groups were compared by the *t*-test, whereas those among several groups were compared by one-way ANOVA. *p*< .05 suggested that the difference was statistically significant.

## 3. Results

### 3.1. Characterization of exosomes and EXO-DTX

**Figure 2.**
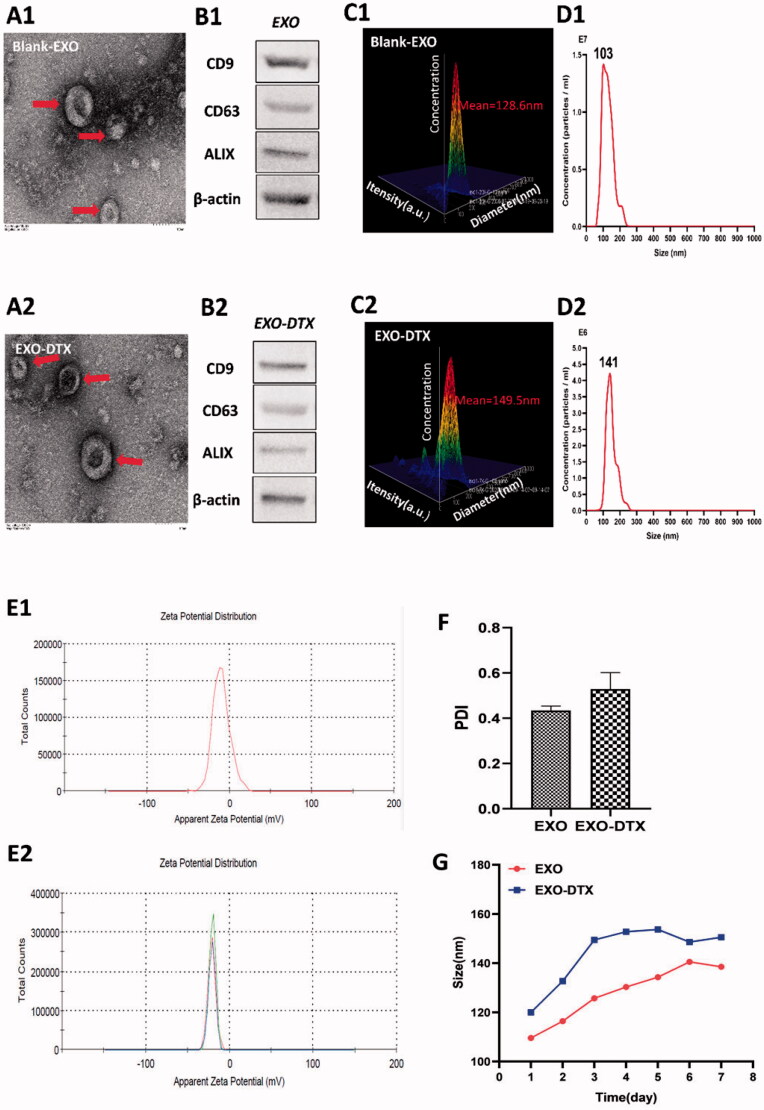
Characterization of the EXO and EXO-DTX. (A1, A2) Scale bar represented 100 nm. Morphology of EXO and EXO-DTX under transmission electron microscopy. (B1, B2) Western blot (WB) analysis of EXO and EXO-DTX. (C1, C2) The average size of the EXO and EXO-DTX group. (D2, D2) The micelle size distribution in the EXO and EXO-DTX group. (E1, E2) The zeta potential of EXO and EXO-DTX at 4 °C. (F) The PDI of EXO and EXO-DTX at the 4 °C. (G) The size variation of EXO and EXO-DTX at the 4 °C over time.

As shown in Figure 2, TEM shows that both empty exosomes and EXO-DTX were typically cup-shaped structures. NTA indicated the size of the exosomes to be in a range of 50–250 nm with an average diameter of 128.6 nm and a peak (mode) diameter of 102.1, whereas the EXO-DTX ranged between 94 and 250 nm with a mean diameter of 140.2 nm, and a peak (mode) diameter of 149.5 nm. The size of EXO-DTX did not change significantly compared to the empty exosomes, indicating successful loading of the DTX. Exosomal markers (CD63, CD9, and ALIX) were detected in the exosomes by the WB analysis. The zeta potential of EXO and EXO-DTX was −9.62 mV and −21.1 mV, respectively. The PDI of EXO-DTX was wider than the EXO, indicating that aggregation occurred in the EXO-DTX. During the one week of the stability measurement of EXO and EXO-DTX, the particle size of EXO and EXO-DTX did not change much.

### 3.2. Drug-loading and *in vitro* release study

**Figure 3.**
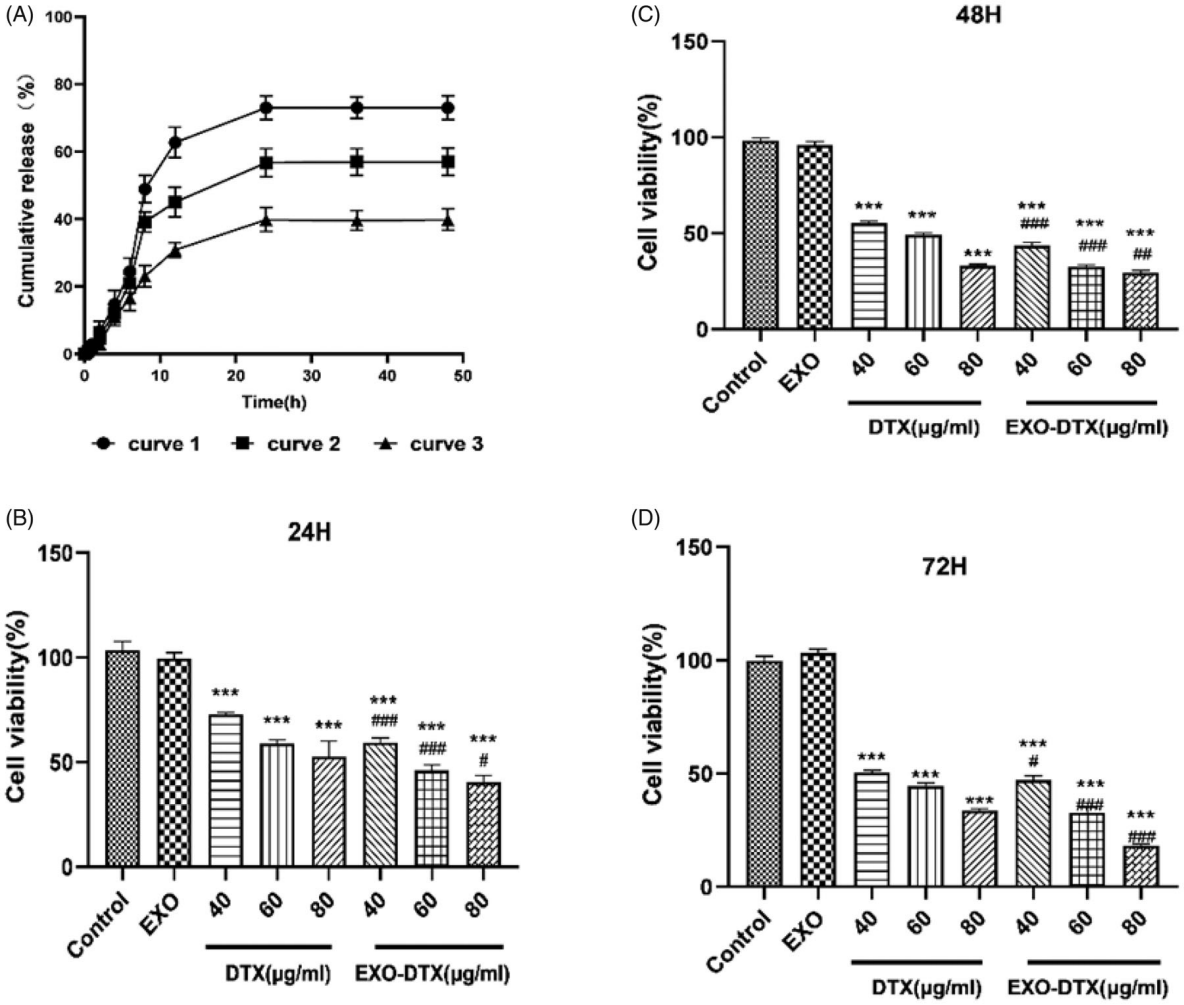
(A) *In vitro* release profiles of DTX and EXO-DTX. Curve 1: DTX release in 1% SDS PBS (pH = 7.4, 37 ± 1 °C); curve 2: EXO-DTX release in 1% SDS PBS (pH = 5.8, 42 ± 1 °C); curve 3: EXO-DTX release in 1% SDS PBS (pH = 7.4, 37 ± 1 °C. (B–D) Cytotoxic effects of DTX and EXO-DTX *in vitro*. After the incubation of the 96-well plate, A549 cells were treated with DTX and EXO-DTX at different doses for 24/48/72 h, respectively. CCK-8 assay was conducted to measure cell viability. Values were expressed as mean ± SD. ***p< 0.001 compared to the control, ^#^*p*< 0.05, ^###^*p*< 0.001 compared to the DTX group at an identical dose.

The EE and drug-loading of DTX in exosomes were found to be 12.2 ± 5.5% and 11.31 ± 4.67%. As seen in the figure, the cumulative release rate of DTX in the normal body fluid environment was found to be 14.9% at 4 h and 73.4% at 48 h, while the cumulative release of EXO-DTX in the normal body fluid environment was found to be 10.4% at 4 h and 40.58% at 48 h, while the cumulative release of EXO-DTX at the tumor site was 12.5% at 4 h and 56.9% at 48 h (Figure 3(A)). The results showed that the EXO-DTX had a significantly slower release in normal body fluid and tumor environment than that of the DTX. Also, the release rate of EXO-DTX in the tumor environment was faster than that found in a normal body fluid environment.

### 3.3. *In vitro* anti-cancer activity

#### 3.3.1. *In vitro* cytotoxic effect

Compared to the control, all the treatments suppressed A549 cell proliferation depending on the time and dose. Also, the tumor cell viability was increased in the DTX-treated group compared to the EXO-DTX-treated cells (Figure 3(B–D)). Therefore, it could be speculated that the exosomes could be a promising DDS.

#### 3.3.2. The influence of exosomes on apoptosis of A549 cells

**Figure 4.**
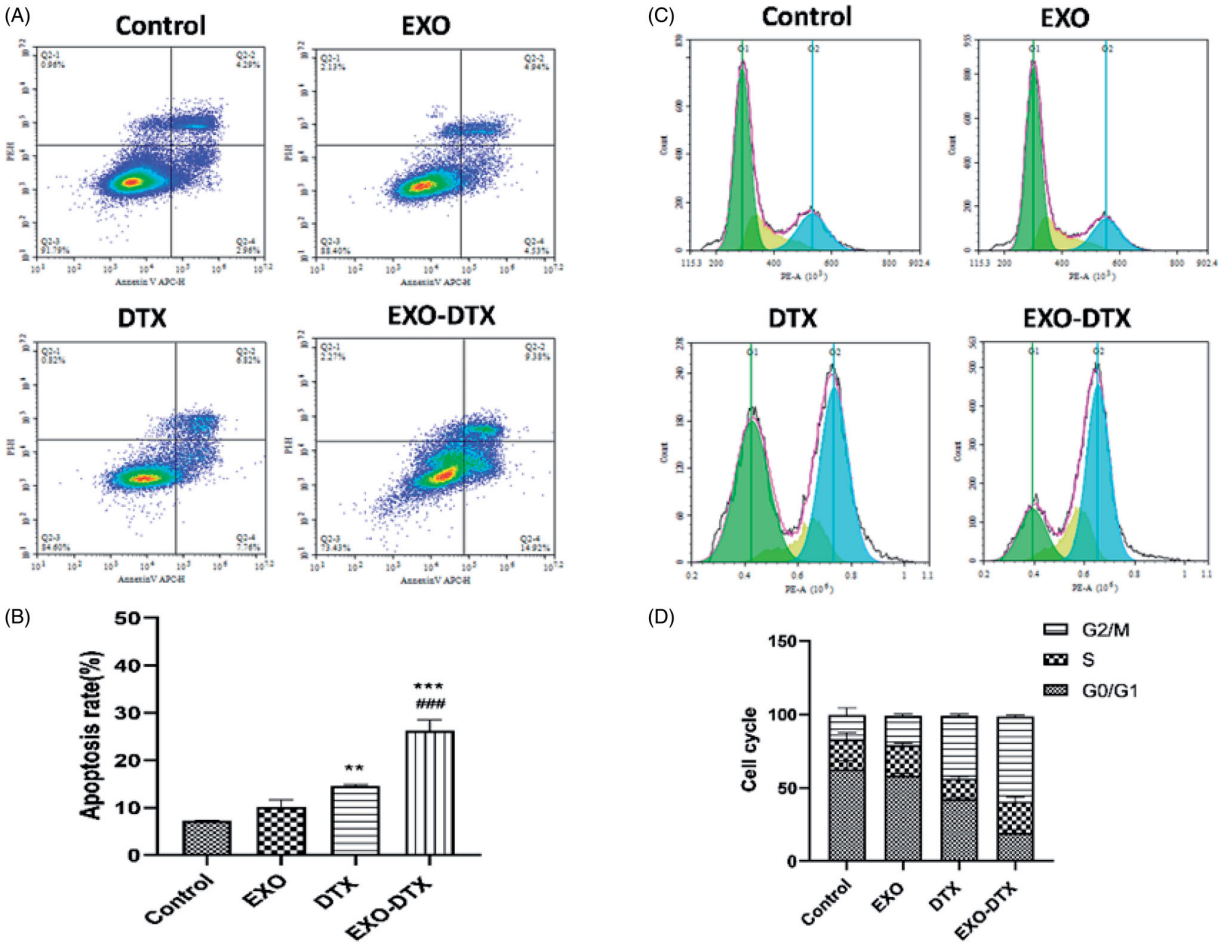
Cell apoptosis analysis *in vitro* (A, B) Analysis of the cell cycle (C, D). After 24 h of DTX, EXO, and EXO-DTX treatment, A549 cells were collected for the Annexin V-FITC/PI staining and FACS, where the A549 cells were collected, fixed by 70% ethanol, and then stained with PI. Finally, each sample was subjected to FACS. Values were expressed as mean ± SD. **p< 0.01, ***p<0 .001 compared to the control, and ^###^*p*< 0.001 compared to the DTX group.

The results indicated that both free DTX and EXO-DTX induced cell apoptosis, but the latter had a significantly increased effect than the former, especially in inducing the late apoptosis, which was consistent with the result of the cell cycle experiment. Thus, the EXO-DTX caused an obvious cytotoxic effect on the A549 cells via the apoptosis pathway (Figure 4(A,C)).

#### 3.3.3. The cell cycle assay

The flow cytometry displayed A549 cell proportions in diverse cell cycle stages (G0/G1, S, and G2/M) after being treated with corresponding drugs. The results showed that the cell proportion at G0/G1 phase in the EXO-DTX group showed a marked decrease compared to the DTX group, while the cells at the G2/M phase in the EXO-DTX group showed an evident increase compared to the DTX group, which indicated that EXO-DTX treatment promoted the arrest of the cell cycle at G2/M phase. Differences in the cell proportion at the S phase were not statistically significant among the three groups, and the survival rate of the mice in the EXO-DTX treatment was found to be lower than that of the DTX treatment group (Figure 4(B,D)).

#### 3.3.4. Cell migration inhibited by the EXO-DTX

Transwell migration assay was chosen to evaluate cancer treatment by EXO-DTX. The results showed that the DTX and EXO-DTX inhibited cell migration. The EXO-DTX group was more significant, which indicated that the EXO-DTX inhibited the tumor by inhibiting cell migration (Figure 4(A,B)).

#### 3.3.5. ROS-mediated apoptosis induced by EXO-DTX

**Figure 5.**
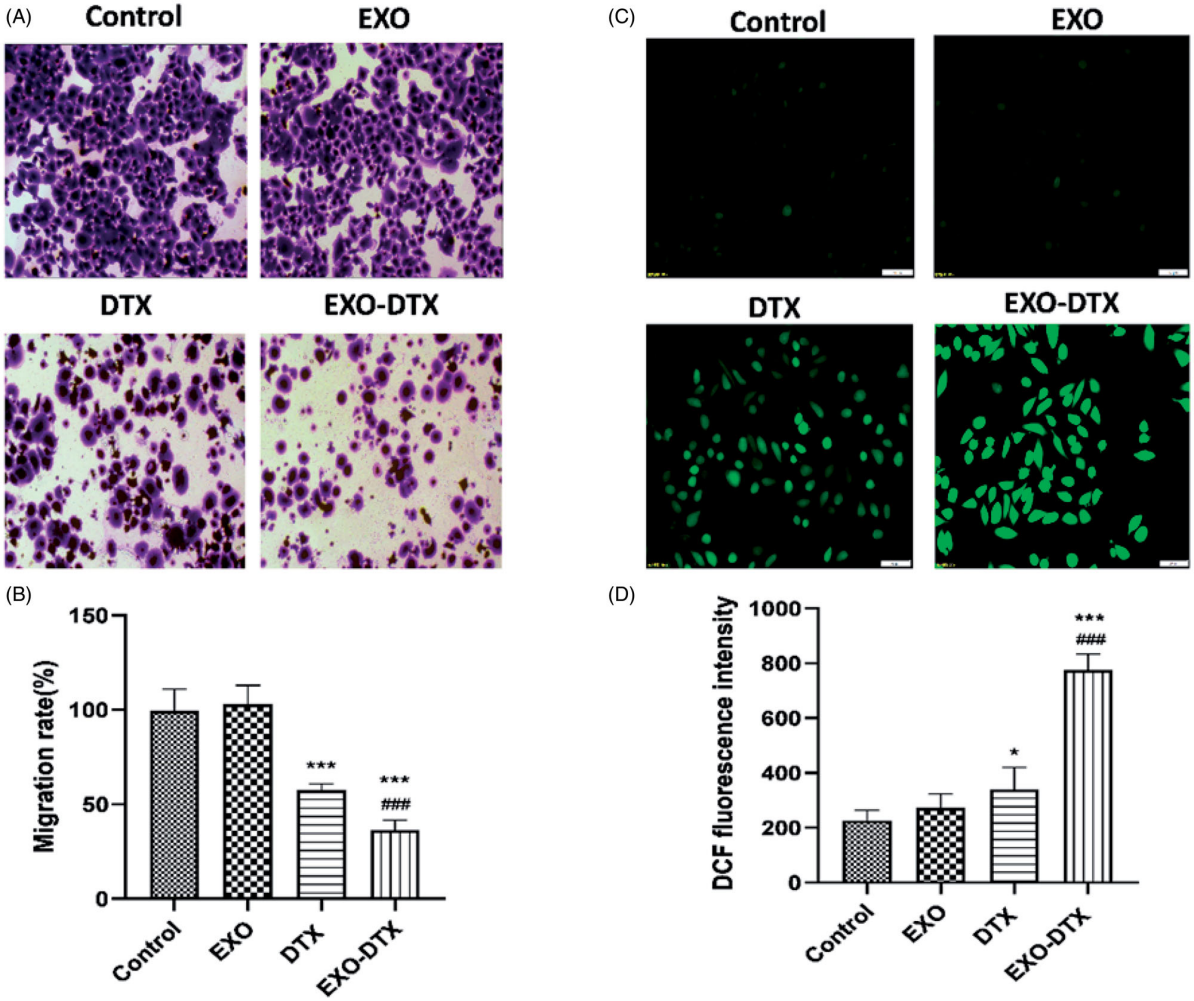
*In vitro* cell migration studies (A, B). ROS production in A549 cells incubated with EXO, DTX, and EXO-DTX (C, D). A549 cells treated with EXO, DTX, and EXO-DTX were stained with DCFH-DA and observed under fluorescent microscope (×20). Scale bar represented 50 μm. ***p< 0.001; *p<0 .05 vs. control; ^###^*p*<0 .001 vs. the DTX group.

FACS was used to detect the roles of DTX and EXO-DTX in ROS production in the A549 cells. DTX was also shown to promote ROS generation in A549 cells, but the more obvious effect was detected in the EXO-DTX group (Figures 4(C,D) and 5).

### 3.4. Cellular uptake assay

**Figure 6.**
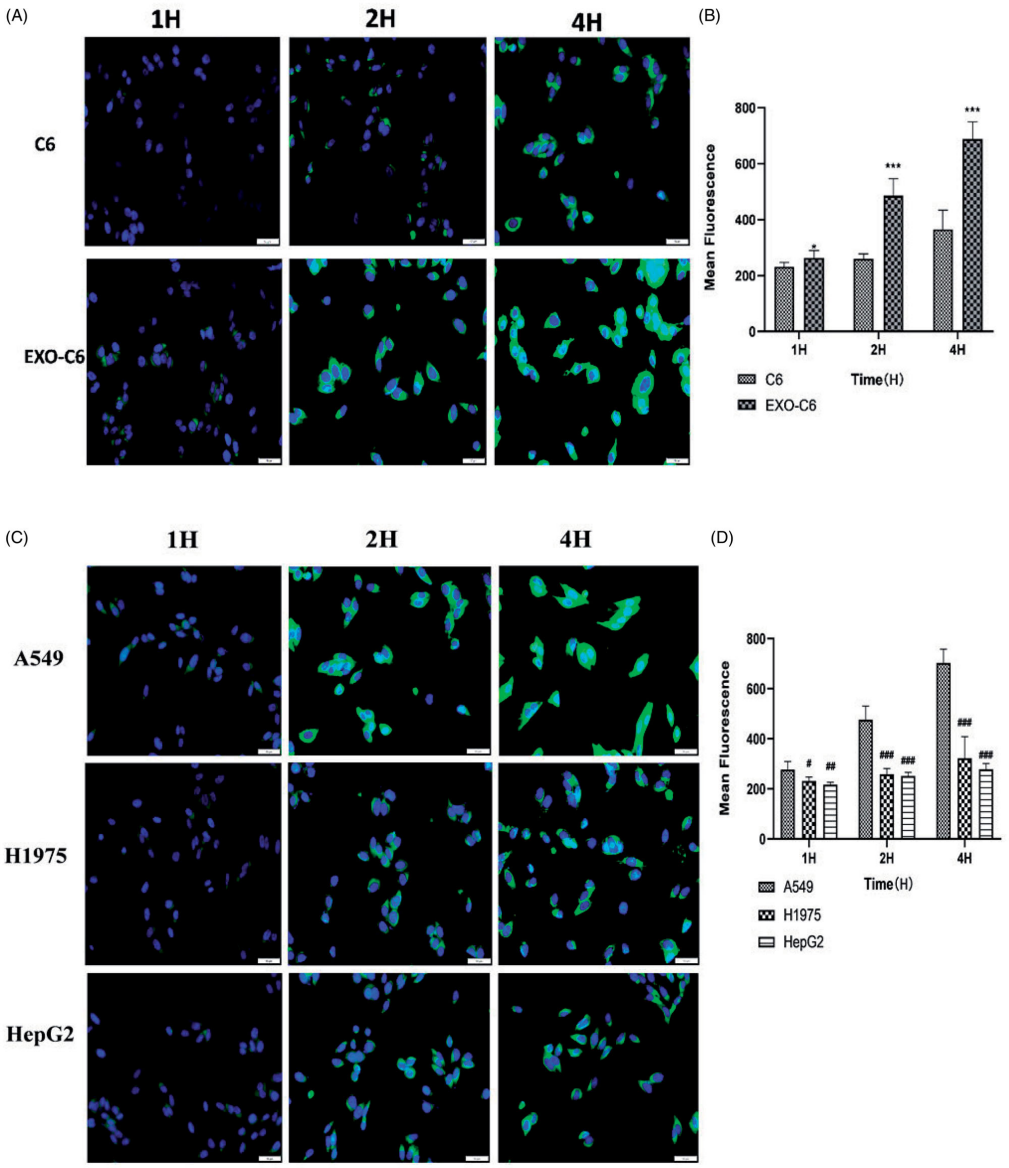
(A) Fluorescence microscopy images of C6 and EXO-C6 after incubating A549 cells for 1, 2, and 4 h. (B) A statistical analysis diagram showing the fluorescence intensities of C6 and EXO-C6. (C) Fluorescence microscopy images of EXO-C6 after incubating A549, H1975, and HepG2 cells for 1, 2, and 4 h. (D) A statistical analysis diagram showing the fluorescence intensities of EXO-C6. Values were displayed as mean ± SD (*n* = 3). Scale bar represented 50 μm. ***p<0 .001 vs. the C6 group. ^#^*p*<0 .05 vs. the A549 group, ^##^*p*<0 .01 vs. the A549 group, and ^###^*p*< 0.001 vs. the A549 group.

To observe the cellular uptake of EXO, A549 cells were incubated with free C6 and EXO-C6 for 1, 2, and 4 h. Cells treated with EXO-C6 showed higher fluorescence signal compared to the C6 group, suggesting an easier uptake of EXO-C6 by A549 cells. The results displayed that the cellular uptake of EXO-C6 by A549 cells was time-dependent. Besides, to compare the autologous and heterologous cellular uptake of exosomes, A549 cells, H1975 cells, and A549 cells were cultured. The results displayed that both autologous (Figure 6(A,B)) and heterologous (Figure 6(C,D)) uptake were time-dependent; however, the autologous uptake efficiency was significantly higher than heterologous uptake, indicating that exosomes prefer the autologous tumor cells.

### 3.5. *In vivo* targeting of EXO-DTX

**Figure 7.**
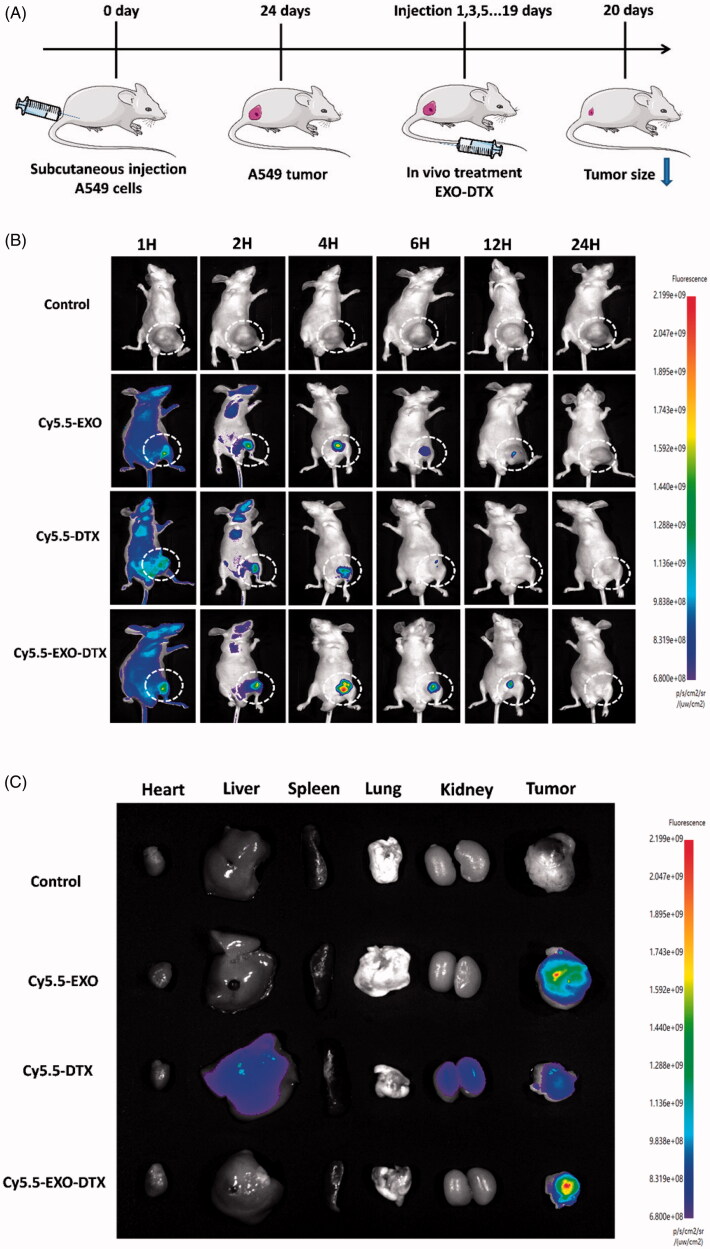
(A) Schematic graph of the experimental design. (B) Fluorescence distribution of saline, Cy5.5-EXO, Cy5.5-DTX, and Cy5.5-EXO-DTX at various time points. (C) Fluorescence signal distribution in the isolated organs.

The results of EXO-DTX treatment revealed a weak fluorescence signal in tumor tissues of mice, suggesting EXO-DTX’s ability to efficiently target tumors. One hour, 2 h, 4 h, 6 h, 12 h, and 24 h time points were chosen to record results in our study. We found that after 4 h of injection, the signal reached a maximum. Conforming to the imaging results *in vivo*, a marked increase was detected in the fluorescence signal from the EXO-DTX-treated tumor tissues compared to that of the free DTX treated tissues. Therefore, the EXO-DTX displayed a better antitumor effect, possibly because of its efficient and rapid accumulation within the target tumor tissues at tumor sites. Also, the biodistribution of DTX in the major organs and tumor was recorded, and EXO-DTX was found to enhance the targeting efficiency. A549 tumor absorbed much more EXO-DTX compared to just the DTX. The data demonstrated that the EXO-DTX effectively accumulated and released DTX at the tumor site of A549, thus, showing a higher tumor-targeting ability in the mice tumor model with more potential than DTX. Overall, EXO-DTX showed superior efficacy over DTX under the *in vivo* conditions (Figure 7).

### 3.6. *In vivo* anti-cancer activity of EXO-DTX

**Figure 8.**
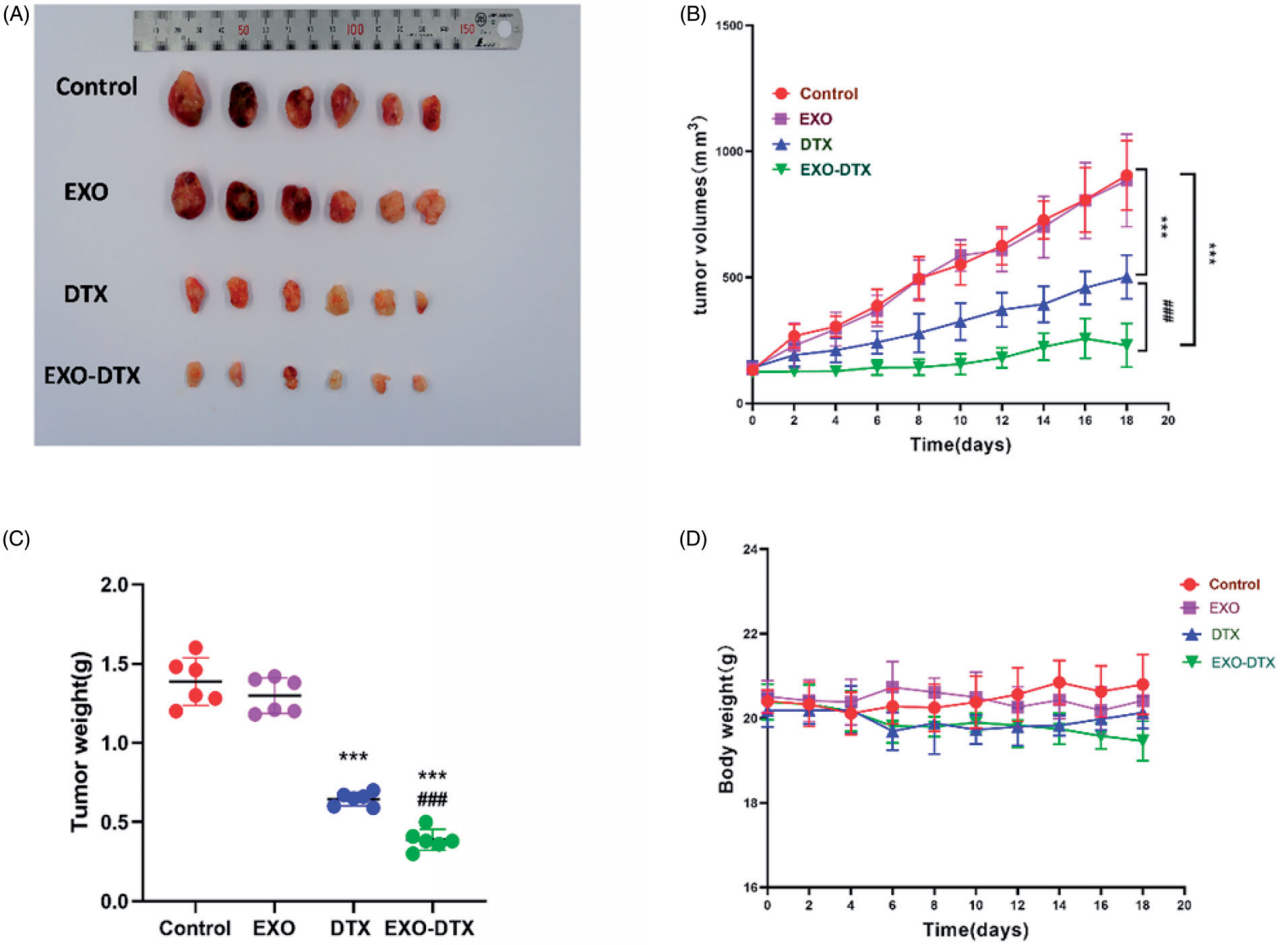
Antitumor activity *in vivo*. Mice (*n* = 6) were given 19 days of oral administration of DTX, EXO, and EXO-DTX. (A) After anesthesia, tumor tissues were resected from mice for imaging purposes. (B) Tumor tissues were monitored at an interval of two days. ***p< 0.001 compared to control, ^###^*p*< 0.001compared to the DTX group. (C) Tumor weight. ***p< 0.001 compared to control, ^###^*p*< 0.001 compared to the DTX group. (D) The body weight. Values were displayed as mean ± SD. **p< 0.01, ***p< 0.001 compared to control, ^##^*p*< 0.01, ^###^*p*< 0.001 compared to the DTX group (4 mg/kg).

The tumor size and weight of the mice treated with Cy5.5-EXO-DTX were found to be reduced compared to that of the mice exposed to identical Cy5.5-DTX treatment (Figure 8(A–C)), which revealed that the EXO-DTX was an efficient method to suppress tumor development in the A549 model.

Consistent with the *in vitro* imaging results and compared to the control group (NS), the accumulation of Cy5.5-DTX and Cy5.5-EXO-DTX in the tumor sites were time-dependent, while the fluorescence signals detected from the tumor in the Cy5.5-EXO-DTX treatment showed a marked increase compared to that found in the Cy5.5-DTX treatment, which indicated that the Cy5.5-EXO-DTX preferred the tumor tissue. The tumor size in mice treated with the Cy5.5-EXO-DTX showed a decrease compared to those that belonged to the identical Cy5.5-DTX treatment group, suggesting that the EXO-DTX was an efficient method to suppress tumor development in the A549 model.

### 3.7. Histological (HE) analysis

**Figure 9.**
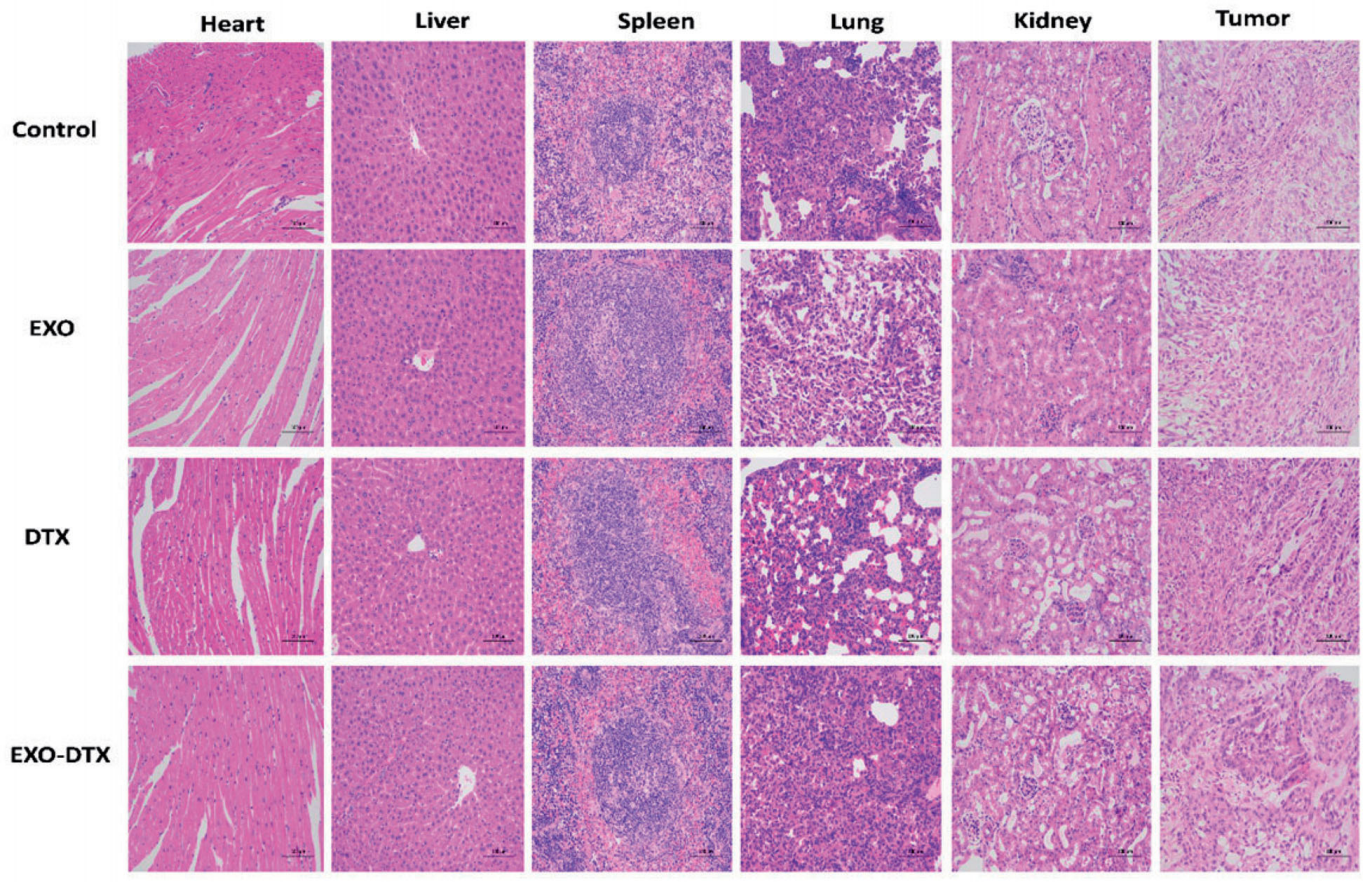
H&E staining images of tumor-bearing mice heart, liver spleen, lung, kidneys, and tumor (×200).

Simultaneously, H&E staining was also conducted on the organs of mice, including hearts, livers, spleens, lungs, kidneys, and tumors (Figure 9).

## 4. Discussion

NSCLC, one of the most life-threatening lung cancer, is the leading cause of cancer related deaths at a global level (Salehi et al., 2020). All available drugs for lung cancer have side effects and are less effective against NSCLC (Fan et al., 2017). Therefore, there is an urgent need to discover a safe and effective DDS for NSCLC treatment. DTX is one of the most important anti-cancer drugs and has widely been used in the treatment of various types of cancer, such as ovarian cancer, lung cancer, and liver cancer. However, the clinical treatment of DTX has been greatly limited (Zhang et al., 2019; Wang et al., 2020) due to its low water solubility and poor selectivity of tumor cells for injection. Therefore, it is urgently required to develop alternative strategies for the treatment of NSCLC. In this context, nanotechnology seems to be the most promising strategy. Multiple drugs can be co-loaded nanoparticulate DDS that could release the drug in a controlled manner. This approach can help in targeting tumor sites through the enhanced permeability retention (EPR) effect, leading to overcome the limitations associated with the conventional combination therapy. Recently, some studies have indicated that exosomes can be used as carriers to carry anti-cancer drugs (Meng et al., 2020). Especially cancer cell-derived exosomes (Emam et al., 2019), i.e. autologous exosomes, were preferentially accumulated within tumor part compared to allogeneic exosomes, which showed good targeting property. Based on the results of previous studies, it would be interesting to use A549 tumor cells exosomes as a carrier and study their role in antitumor DDSs.

First, exosomes were isolated by ultracentrifugation. DTX was loaded into exosomes using electroporation. The results of TEM and NTA showed that DTX was loaded into exosomes successfully. The PDI results of EXO and EXO-DTX indicated that the particle size difference in the system is small and the particle dispersion is uniform; the uniformity of the system is good. The stability results indicated that both EXO and EXO-DTX could be stable within one week under the condition of 4 °C.

The EE and drug-loading reached 12.2 ± 5.5% and 11.31 ± 4.67%, respectively.

Furthermore, we investigated the *in vitro* release of EXO-DTX. The result showed that the drug release performance of EXO-DTX was slower than DTX, indicating that EXO-DTX had better sustained-release property than free DTX. It may be because of the bilayer structure of exosomes that the drugs have the function of sustained release, making the drug in the body with the function of slow release, prolong the release time of the drug in the body, enhance the efficacy, which is consistent with previous literature reports (Kim et al., 2016; Li et al., 2020).

The *in vitro* antitumor activity showed that EXO-DTX could effectively inhibit proliferation, promote apoptosis, disturb the cell cycle, and inhibit the migration of A549 tumor cells. In the experiments of cytotoxicity, we found that the inhibiting effect of EXO-DTX was much better than that of DTX, EXO-DTX can effectively disturb the cell cycle, cause cell metabolism disorder, and promote apoptosis, which may be because exosomes can effectively fuse with cells to promote the absorption of drugs and exert their efficacy (Betker et al., 2019). That is, tumor-derived exosomes with tumor homing properties can efficiently reach cancer cells and can therefore act as carriers to improve drug delivery to primary tumors and metastases (Vázquez-Ríos et al., 2019). Cell migration assay is of great guiding significance for the condition of tumor cells (Qiang et al., 2019). The results of transwell assay indicated that the EXO-DTX could significantly inhibit the A549 migration compared with DTX, while the blank exosomes same as the control group has no inhibition of migration. In recent years, an increasing number of reports have stated that some natural products can induce tumor cell ROS accumulation and ultimately kill tumor cells (Haugrud et al., 2014). We found that EXO-DTX can induce the generation of ROS in A549 cells. In other words, the EXO-DTX could destroy the metabolic process, play a therapeutic effect by regulating the ROS content in cells. Besides, cellular uptake study demonstrated that the internalization of EXO-C6 in A549 cells was higher as compared to the free C6; cellular uptake study of A549 cells, H1975 cells, and HepG2 cells also confirmed a comparatively increased efficacy of cellular uptake of autologous exosomes, it may be that the membrane of exosomes is similar to the mother membrane and has a strong affinity to promote the absorption of drugs, suggesting that exosomes can show preference to their parental cells (Zhang et al., 2020).

Finally, in view of the significant efficacy of the above *in vitro* cell efficacy, the efficacy of the animal *in vivo* was then investigated and the *in vivo* efficacy of Cy5.5-EXO-DTX in A549 tumor-bearing nude mice was determined. The administration of Cy5.5-EXO-DTX inhibited the tumor growth significantly and caused less change in body weight of the tumor-bearing mice in comparison with the free Cy5.5-DTX group which indicates that exosomes as carriers have good safety for injection and will not produce toxic and side effects on animals. *In vivo* imaging experiments that Cy5.5-EXO-DTX group had the strongest fluorescence, followed by Cy5.5-EXO, and Cy5.5-DTX group had the weakest fluorescence, while the NS group had no fluorescence. It was possible that Cy5.5-EXO-DTX not only had the active targeting effect of exosomes, but also had the EPR of the tumor tissue itself, which prolonged the retention time of the drug in the tumor tissue. They both enhance the targeting effect of Cy5.5-DTX-EXO. Cy5.5-EXO reaches the tumor site by active targeting of exosomes. Cy5.5-DTX can only reach the tumor site by EPR effect (Golombek et al., 2018; Dhaliwal & Zheng, 2019). In addition, Cy5.5-DTX group was distributed in both liver and kidney tissues. This indicates that this group lacks targeting, and the drugs are distributed randomly in the body. Therefore, under the targeting effect of exosomes and the effect of EPR, drugs can better target the tumor site and exert a therapeutic effect. From the H&E staining of major organs, it could be found that there were slight voids appearing in the cytoplasm of the liver, lung and kidney in each administration group, especially in the lung, this may be related to the high metastasis of A549 tumor cells (Huang et al., 2018), while from the H&E staining of tumor, the Cy5.5-EXO-DTX presented better antitumor efficacy in A549 tumor-bearing mice models than Cy5.5-DTX group. This may be due to the targeting of drugs after the inclusion of exosomes, which can guide the specific delivery of drugs to tumor tissues, improve the efficacy. Thus, we strongly believe that our research provides a reference for the further development of targeted delivery drugs for the treatment of NSCLC.

## 5. Conclusions

In our study, tumor-cell exosomes were chosen for drug delivery. As revealed by the experimental results *in vitro*, EXO-DTX markedly suppressed the A549 cell proliferation while increasing the cytotoxic effect *in vitro*. Also, FACS analysis indicated that the EXO-DTX possibly promoted apoptosis, induced cell cycle arrest at the G2/M phase, increased the ROS generation, and ultimately displayed the anti-cancer effect. The cell uptake assays also showed high cell uptake. Moreover, the experimental results *in vivo* demonstrated that the EXO-DTX could be used as the tumor-targeting agent with a sustained-release formulation that has an increased drug potency compared to that of the free DTX. Therefore, we conclude that the exosomes could be a potential DDS in providing an effective antitumor activity of DTX.

## Supplementary Material

Supplemental Material
