# Supplementary figures and images for: Docetaxel-loaded exosomes for targeting non-small cell lung cancer: preparation and evaluation in vitro and in vivo
Source: Drug Deliv. 2021 Jul 15;28(1):1510–23. doi: 10.1080/10717544.2021.1951894 (PMC8284156; doi:10.1080/10717544.2021.1951894)

**Fig.2**


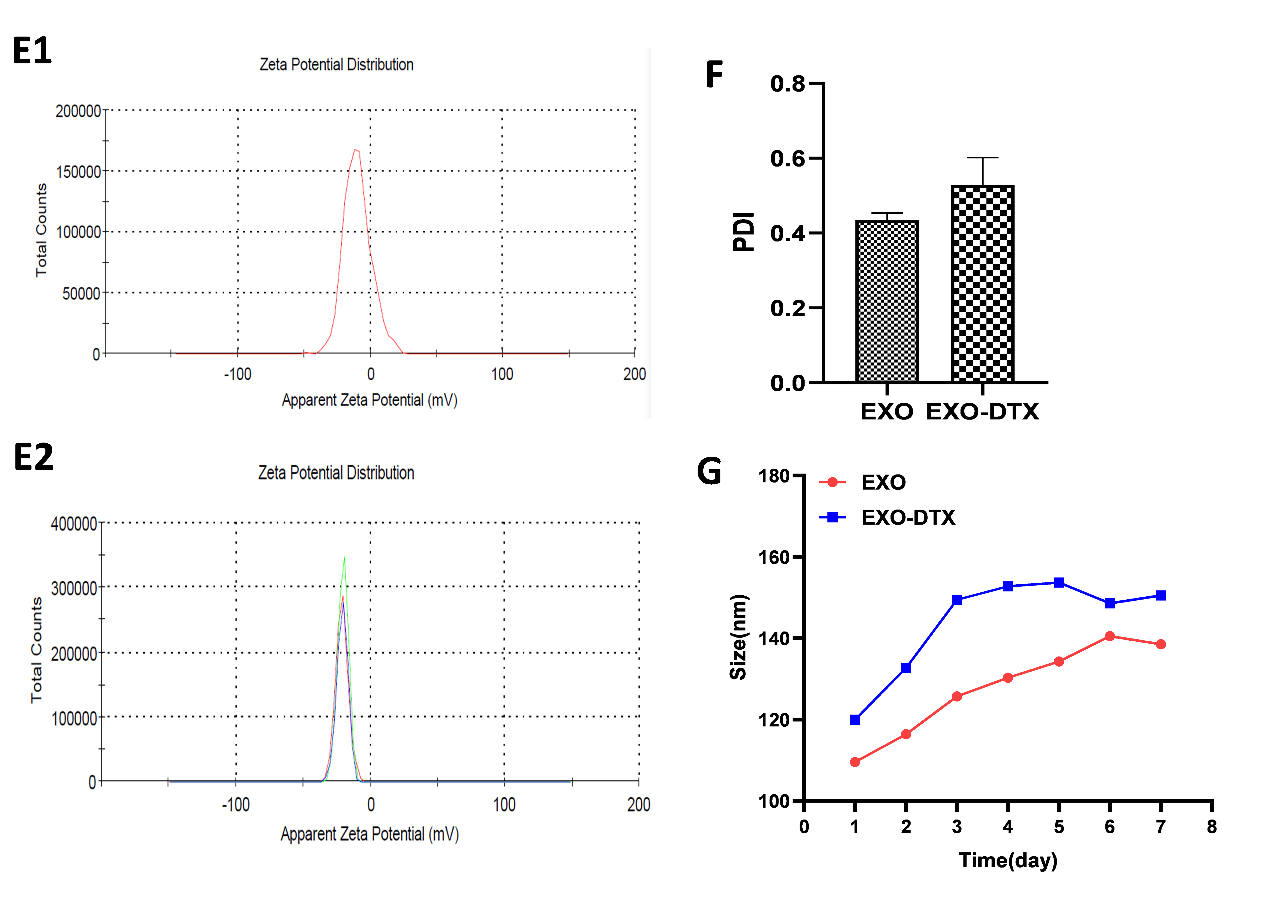


**Fig.6**


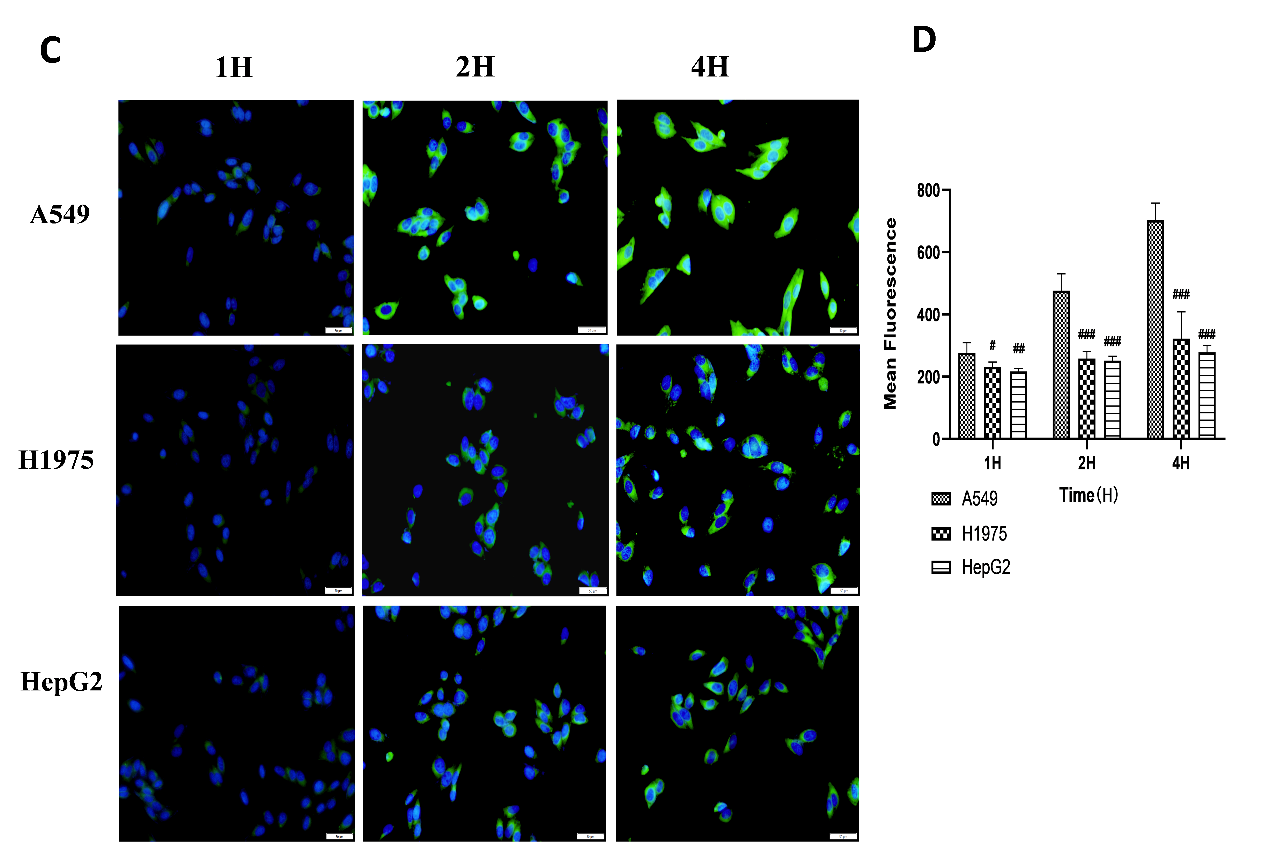

Supplement: Supplemental Material [file IDRD_A_1951894_SM7806.docx]
